# Clozapine Toxicity in the Setting of COVID-19: A case of differential diagnosis

**DOI:** 10.1192/j.eurpsy.2022.1298

**Published:** 2022-09-01

**Authors:** J.M. Pelayo-Terán, Y. Zapico-Merayo, S. Vega-García

**Affiliations:** Hospital El Bierzo. GASBI. SACYL, Psiquiatría Y Salud Mental, Ponferrada, Spain

**Keywords:** clozapine, Covid-19, pneumonitis, fever

## Abstract

**Introduction:**

Together with agranulocytosis, fever and immflamatory manifestations are clozapine side effects to be monitorized during initial treatment. In the context of COVID-19 pandemic, implied mechanisms, and symptomatology should be carefully controlled.

**Objectives:**

To analyze the clinical analytic and inflammatory chracterisctics the resembles and differenciates clozapine immune response and SARS-CoV-2 infection. To describe a case of clozapine induced fever and pneumonitis during COVID-19 pandemic.

**Methods:**

A case of clozapine-induced pneumonitis during COVID-19 pandemic is described.- A mini-review of clozapine inflamamtory effects, induced-pneumonitis and SARS-CoV-2 was performed.

**Results:**

A 33 year old afrolatin male started treatment with clozapine up to 250 mg daily. He developed fever and respiratory symptoms in the 11th day of treatment. The exploration revealed pulmonary sounds decreased and 91% basal saturation, making the probable causes viral infection (local incidence of SARS-CoV-2 >800/100000hab), nosocomial bacterial infection or pulmonary thromboembolism. The patient was isolated due to probable COVID-19. Blood tests showed leucocytosis (13400/mcL), Lymphocytopenia (11.8%), high PCR (14.4mg/dL), Ferritine (506.9ng/mL), Fibrinigen (663.83 mg/dL), D-Dimer (1.61mg/dL), and Interleukin-9 /25.8pg/mL). The angioTC revealed a pleural efusion and ground glass infiltrates (figure1). Only after 2 weeks eosinophilia was discovered (88/mcL) After 2 negative consecutieve PCRs for SARS-CoV-2, no imrovement with ampirical antibiotics and all infectious pannels negative, we started decreasing clozapine with improvement of the symptoms and resolution after suspending clozapine completely.

**Figure:**
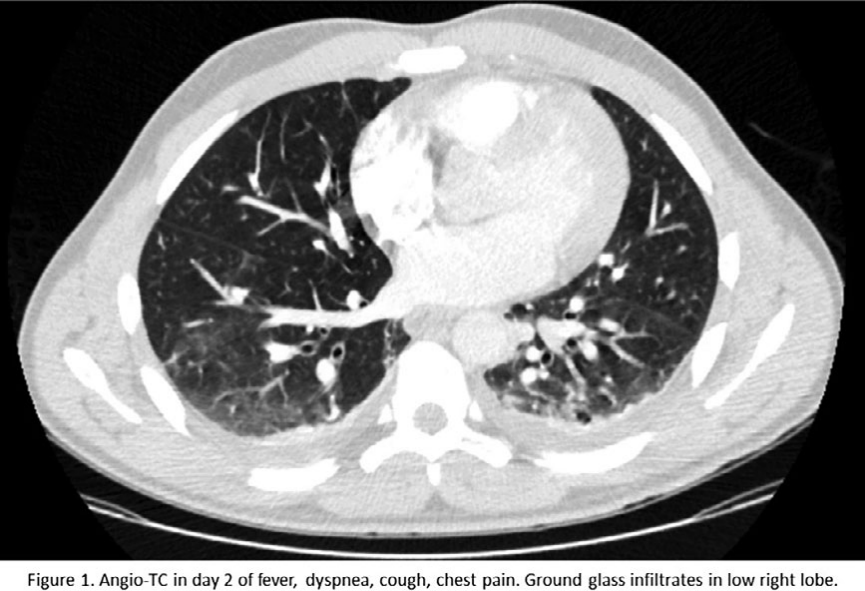

**Conclusions:**

Clozapine may induce a generalize inflammatory response mediated by interleukin-6. Patients treated with clozapine may exhibit fever and rarely, insterstitial lung inflammation. The expression of induced pneumonitis resembles viral infections, particularly SARS-CoV-2

**Disclosure:**

No significant relationships.

